# Local synthesis and function of neuro-estrogen and neuro-androgen: impact on the hippocampus-related cognition and neuronal plasticity

**DOI:** 10.3389/fncel.2025.1695565

**Published:** 2026-01-28

**Authors:** Suguru Kawato, Yasushi Hojo, Mika Soma, Shigeo Horie, Minoru Saito, Mari Ogiue-Ikeda

**Affiliations:** 1 Department of Biophysics and Life Sciences, Graduate School of Arts and Sciences, The University of Tokyo, Tokyo, Japan; 2 Department of Cognitive Neuroscience, Faculty of Pharma-Science, Teikyo University, Tokyo, Japan; 3 Department of Urology, Graduate School of Medicine, Juntendo University, Tokyo, Japan; 4 Department of Biosciences, College of Humanities and Sciences, Nihon University, Tokyo, Japan; 5 Department of Biochemistry, Faculty of Medicine, Saitama Medical University, Saitama, Japan; 6 Department of Anatomy and Neurobiology, Nippon Medical School, Tokyo, Japan

**Keywords:** aging, androgen, cognition, estrogen, hippocampus, spine, synaptic plasticity

## Abstract

Brain sex neurosteroids have been attracted much attention, because the brain itself can synthesizes a sufficient amount of sex neurosteroids independent of circulating sex steroids. Local synthesis and action of neuro-estrogen [such as neuro-estradiol (nE2)] and neuro-androgen [such as neuro-testosterone (nT) and neuro-dihydrotestosterone (nDHT)] have become recognized as key factors in modulation of synaptic plasticity, cognitive performance, and protection of aging dependent decline of neurological functions. Unlike circulating sex steroids, these locally synthesized sex neurosteroids can directly and rapidly modulate neuronal synapses and induce potent effects on learning and memory. The properties of local neurocrine systems are significantly different from those of classical neuroendocrine systems dependent on the hypothalamic–pituitary-gonadal axis. For example, in the hippocampus, not only neuro-androgen (nT and nDHT) but also nE2 have higher concentrations than testis-derived circulating androgen (T and DHT) and ovary-derived circulating E2 (cir-E2). In addition, male nE2 concentration is higher than female nE2 at both adult stage and newborn stage during which brain masculinization occurs. Over the past decades, numerous experimental results and interpretations of sex neurosteroids have been shown. However, in several cases, these results and interpretations are mutually conflicting, and a unified understanding has not yet been achieved. Therefore, we here deeply discuss several critical and important issues toward solving complex problems to understand. We focus the following issues. First (A) Local synthesis of nE2, nT and nDHT, with particular attention to their concentrations, synthesis pathways and sex differences in rodents. Higher nE2 concentration in male hippocampus than in female in adult stage. Then (B) Comparison of modulation of long-term potentiation (LTP) by nE2 and nDHT. Stimulatory effects of nE2 on LTP which are mediated by membrane estrogen receptor ER and protein kinase signaling. Inhibitory effects of nDHT on LTP which are mediated by membrane androgen receptor AR. Third (C) Both nE2 and nDHT show the same rapid increases in dendritic spines. Their effects on spinogenesis are very different from their effects on LTP. Fourth (D) Rapid effects of nE2 and nT on cognitive behavior. Male signaling pathway may be more complex than female signaling pathway. Finally (E) Aging-dependent cognitive decline which is dependent on decrease of nT in male and nE2 in female. T-replacement therapy of male patients shows improvement in spatial cognitive decline. E2-replacement therapy improves female cognitive decline.

## 1 Introduction

For decades, extensive studies have been performed to analyze the role of 17β-estradiol (E2) and androgen in modulating the hippocampal neural plasticity of rodents, because the hippocampus, center for learning and memory, has been realized to be an important and attractive target for the actions of gonadal estrogen and androgen reaching the brain via blood circulation. The following many important results about their actions have been obtained.

The density of dendritic spines in the CA1 pyramidal neurons is modulated relatively slowly (1–4 days) by supplementation of E2 in ovariectomized (OVX) rodents (Gould et al., 1990; Leranth et al., 2000; Leranth et al., 2002; Woolley et al., 1990; Woolley et al., 1997), resulting in increase/recovery of spines.

Four days after s.c. injection of testosterone (T) and dihydrotestosterone (DHT), the spine-synapse density, observed by electron-microscopic analysis, was increased in hippocampal CA1, and flutamide (AR blocker) suppressed these DHT and T effects in castrated male rats (MacLusky et al., 2004).

*In vitro* investigations have also shown that the spine density is increased following several days’ treatment of cultured hippocampal slices with E2 (Murphy and Segal, 1996; Pozzo-Miller et al., 1999).

The slow/genomic modulation of N-methyl-D-aspartic acid (NMDA) receptors by E2 replacement enhances LTP in OVX rat (Smith and McMahon, 2006).

These slow effects of E2 and androgens are mediated via nuclear estrogen receptors ERα/ERβ which initiate transcription processes.

Importantly, rapid electrophysiological modulation by brief E2 perfusion has also been investigated extensively with acute hippocampal slices (Foy et al., 1999; Bi et al., 2000). However, candidates of estrogen receptors that perform these rapid actions had been very difficult to identify until recently.

**Figure 1:**
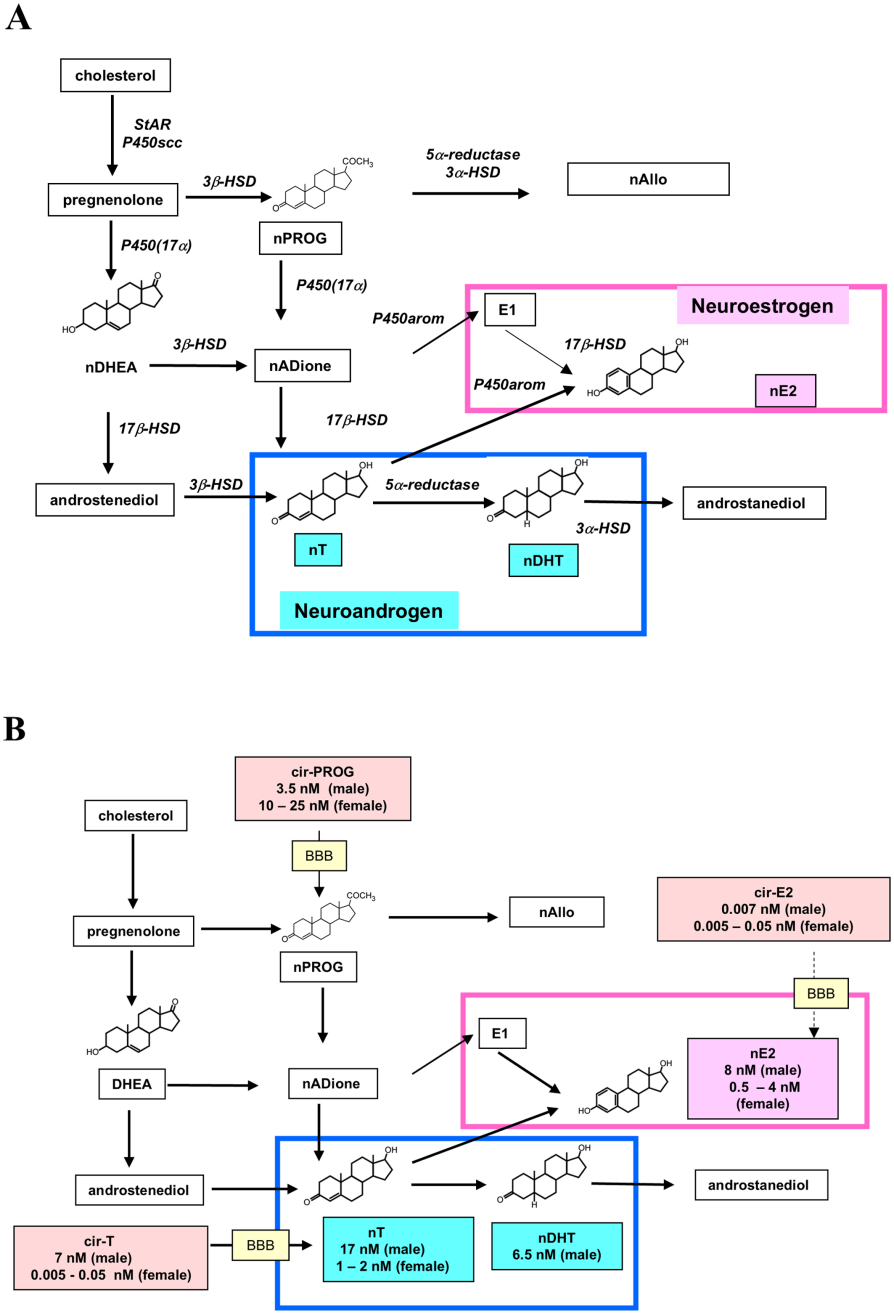
**(A)** Local biosynthetic pathway of nE_2_, nT, and nDHT in rodent hippocampus. Male and female. Abbreviations used are: progesterone (PROG), dehydroepiandrosterone (DHEA), androstenedione (ADione), estrone (E1), allopregnanolone (Allo), circulating E_2_ (cir-E_2_), circulating T (cir-T), circulating PROG (cir-PROG). **(B)** Local synthetic pathway of nE_2_, nT, and nDHT in male and female rat hippocampus is shown with circulating sex-steroids. Hippocampal nT may be a combination of nT and penetrated part of cir-T. Upon castration of male, nT is significantly decreased to 20–30% of intact rat level (Hojo et al., 2009). A very weak contribution of cir-E_2_ to nE_2_ is suggested, because the level of cir-E_2_ is much lower than locally synthesized E_2_. Female nE_2_, cir-E_2_, nT, and cir-T show oscillation along estrous cycle. Some contribution of circulating PROG (cir-PROG) to hippocampal neuro-PROG (nPROG) may exist. Numerical data of concentration of steroids are taken from Hojo et al. (2009) and Kato et al. (2013). Note that the whole blood concentrations of cir-T, cir-E_2_, and cir-PROG are used here, and they are approximately half of plasma concentrations. BBB is the blood brain barrier.

Through the other direction of research, the possibility of endogenous synthesis of neurosteroids [including pregnenolone, progesterone and dehydroepiandrosterone (DHEA)] in the brain was indicated (Baulieu, 1997; Schumacher et al., 1997; Baulieu and Robel, 1998; Kimoto et al., 2001). After several decades of intensive investigations, the whole picture of local synthesis of neuro-estrogen and neuro-androgen was achieved in the adult male/female hippocampus, and these results opened a new field of their functions in relation to the regulation of daily memory formation (Figures 1A,B) (Naftolin et al., 1996; Kawato et al., 2002; Shibuya et al., 2003; Prange-Kiel et al., 2003; Hojo et al., 2004; Kretz et al., 2004).

For practical reasons in this paper, we use the terms neuro-estradiol (nE2), neuro-testosterone (nT) and neuro-dihydrotestosterone (nDHT) to refer to E2, T, and DHT synthesized within the brain (Figures 1A,B).

The nE2 has become recognized as an essential neural modulator of synaptic plasticity and cognitive performance in the hippocampus (Balthazart and Ball, 2006; Mukai et al., 2007; Hojo et al., 2011; Kato et al., 2013; Taxier et al., 2020; Azcoitia et al., 2022; Garcia-Segura et al., 2023). Neuro-androgens (nT and nDHT) also have been recognized as modulators of neural plasticity, however, neuro-androgens differently modulate neurons in comparison with nE2 (MacLusky et al., 2004; Okamoto et al., 2012; Hatanaka et al., 2015; Murakami et al., 2018; Soma et al., 2018).

It is interesting that the levels of nE2, nT and nDHT are higher in the hippocampus than blood plasma/serum (Hojo et al., 2009; Hojo and Kawato, 2018), and therefore it is suggested that circulating estrogen and androgen have a limited role in modulation of brain function. In addition, neuro-allopregnanolone (nAllo) is synthesized from progesterone in the brain including hippocampus (Figures 1A,B), and nAllo elicits anxiolytic, anti-stress and antidepressant effects by facilitating the action of GABA at synaptic GABA_A_ receptors (Paul et al., 2020).

In this paper, we address several important issues focusing on synthesis and function of nE2, nT and nDHT in the adult rodent hippocampus in the following Sections 2–4. In Section 5 we address aging-induced impairment of neurons and cognition due to nT and nE2 decrease, and its rescue by T and E2 supplementation, not only in rodents but also humans (Burke and Barnes, 2006; Morrison et al., 2006; Kawato et al., 2025a; Kawato et al., 2025b).

## 2 Local synthesis of hippocampal nE2, nT, and nDHT, focusing on their concentrations, synthesis pathways, and differences between male and female

Local synthesis of nE2 has attracted much attention and has been extensively studied in rodents and birds (Naftolin et al., 1996; Balthazart and Ball, 2006; Hojo and Kawato, 2018). Through many studies of local synthesis of nE2 and nT from cholesterol in adult rat and mouse hippocampus (Kimoto et al., 2001; Kawato et al., 2002; Shibuya et al., 2003; Hojo et al., 2004; Kretz et al., 2004), nE2 has become recognized as an essential neuromodulator of synaptic plasticity and cognitive performance (Mukai et al., 2007; Hojo et al., 2011; Taxier et al., 2020).

Interestingly, however, several following questions are still left unanswered. For example, although mRNA levels of steroidogenic enzymes are almost the same between male and female hippocampus, why concentrations of nE2 and nT are significantly different between male and female hippocampus? Not only mRNA expression but also immunostaining of steroidogenic enzymes are not significantly different between male and female hippocampus (Hojo et al., 2004; Kimoto et al., 2010; Kato et al., 2013; Hojo et al., 2014).

Therefore, in the following Sections (2.1, 2.2, 2.3) we discuss in order to reach answers of these questions. Figure 1B (flow of synthesis) and Table 1 are useful for deep understanding. Table 1 summarizes representative data about concentrations of nT and nE2 in hippocampus, hypothalamus, birds, and blood circulation.

**Table 1:** T and E2 levels in the adult hippocampus and blood, including hypothalamus.

Group and reference	Male				Female				Sample	Sample purification	Assay	Animal
	T (nM)		E2 (nM)		T (nM)		E2 (nM)			Solvent, column		Age
	Hippo	Blood	Hippo	Blood	Hippo	Blood	Hippo	Blood	Brain region			
Group A: Kawato et al. (2002)			0.67						Fresh hippo tissue (fresh plasma)	Hexane/ethylacetate, C18 column	RIA	Rat adult
Group A: Hojo et al. (2004)			0.59	0.098								
Group A: Hojo et al. (2009)	16.9	7.3	8.4	0.007					Fresh intact (upper) castration (lower)	Hexane/ethylacetate, C18, n-HPLC	LC/MS/MS steroid derivatization	Rat adult
	3.1	0.1	6.9	0.003								
Group A: Kato et al. (2013)					1.2–2.3	0.01–0.05	0.5 (Est)–4.3 (Pro)	0.005 (Est)–0.055 (Pro)	Fresh intact (upper) OVX (lower)	Hexane/ethylacetate, C18, n-HPLC		
							0.5	0.0025				
Group B: Munetsuna et al. (2009a)			8.3						Fresh (pair housed)	Hexane/ethylacetate, C18	EIA	Rat adult
			12.4						Fresh (single housed)			
Group C: Li and Gibbs (2019)	8.86	6.505	0.08						Frozen tissue	n-butylchloride	LC/MS/MS steroid derivatization	Rat adult
					0.25	0.125	0.07	0.01	Frozen OVX tissue (fresh serum)			
Group D: Caruso et al. (2013)	15	6.75	0.15	0	0.6	0	1.1	0.65	Frozen tissue (frozen plasma)	Methanol/aceticacid, C18	LC–MS/MS	Rat adult
Group E: Konkle and McCarthy (2011)			0.4	0.045			0.36	0.035	P3 frozen tissue (frozen plasma)	Diethylether, C18	RIA	Rat P3
			0.05				0.05		P60 frozen tissue			Rat P60
Group E: Konkle and McCarthy (2011)			9	0.045			8.64 (hypo)	0.04	P3 hypo frozen tissue	Diethylether, C18	RIA	Rat P3
			3.6				1.8 (hypo)		P60 hypo frozen tissue			Rat P60
			9.9				1.13 (POA)		2 h POA frozen tissue			Rat 2 h
Group B: Munetsuna et al. (2009b)	9		5						Fresh hippo slice culture	Hexane/ethylacetate, n-HPLC	RIA	Rat slice culture
Group B: Yamazaki et al. (2013)			6								RIA, LC/MS/MS	
Smith et al. (1975)								0.02 (Est)–0.08 (Pro)	(Frozen plasma)		RIA	Rat adult
Group F: Jalabert et al. (2022)			1.6	0.02					POA frozen tissue		LC–MS/MS steroid derivetization	Bird
Group G: Remage-Healey et al. (2012)								0.06	Microdialysis, plasma		ELISA	Bird

Group A = Kawato Lab; Group B = Yamzaki Lab; Group C = Gibbs Lab; Group D = Melcangi Lab; Group E = McCarthy Lab; Group F = Soma Lab; Group G = Schlinger Lab.

Circulating blood steroid levels are written as a half of plasma/serum steroid levels.

P3 hypo means postnatal 3 day hypothalamus. 2 h means 2 h after birth.

Bird data are written as a comparison.

### 2.1 Comparison of nE2 concentration between male and female adult rodents

Using LC–MS/MS determination, it is observed that adult male nE2 concentration (~8 nM) was greater than adult female nE2 concentration (1 ~ 4 nM, depending on estrous cycle) in adult rat hippocampus (in case of fresh tissues without storage at −80 degree) (Table 1) (Hojo et al., 2009; Kato et al., 2013). Here, it is assumed that 1 g wet tissue has an approximate 1 mL volume, and then the dimension is converted from ng/g wet tissue to nM (Hojo et al., 2004; Hojo et al., 2009).

There could be several possible reasons about this sex difference. Penetration of circulating T (cir-T) (~7 nM) to the hippocampus probably contributes significantly to the high concentration of nT (~17 nM) in the male rat hippocampus (Figure 1B; Table 1). This high level of nT can be used for local nE2 synthesis by P450aromatase (P450arom), resulting in nE2 concentration of ~8 nM. Different from male, female nT level is only ~1 nM, and cir-T level is very low (<0.1 nM). Therefore, female nT may also be locally synthesized from neuro-progesterone (nPROG) (20 ~ 50 nM, depending on estrous cycle).

In other publications, however, nearly the same concentration of nE2 (~0.1 nM) was reported in both male and female adult rat hippocampus (in case of frozen–thawed tissues stored at −80 degree until use) (see Table 1; Li and Gibbs, 2019).

Another serious problem is that even concerning only male rodents, the reported nE2 concentrations in the hippocampus are widely distributed between ~3 pg./g tissue and ~2 ng/g tissue (between ~10 pM and ~8 nM) (Table 1) (Hojo et al., 2009; Caruso et al., 2013; Li and Gibbs, 2019; Kawato et al., 2025c). It should be noted that, on the other hand, the reported nT concentrations in male hippocampus are not largely different but being around ~ 3 ng/g tissue (~10 nM) (Table 1) (Hojo et al., 2009; Caruso et al., 2013; Li and Gibbs, 2019; Kawato et al., 2025c).

The reason of the discrepancy in reported nE2 concentrations is not very clear at the moment. One possible reason might be due to the difference in tissue preparation methods of hippocampus, because fresh tissues (without storage at −80 degree) and frozen–thawed tissues (with storage at −80 degree) have been used for determinations (Table 1) (Kawato et al., 2025c).

In order to solve this problem, the comparison between fresh and frozen–thawed hippocampal tissues should be performed under the same conditions in determination of nE2 and nT concentrations (Hojo et al., 2009; Kawato et al., 2025c). Upon comparison, much lower nE2 concentration was observed in frozen–thawed tissues than nE2 in fresh hippocampal tissues, in addition surprisingly nT concentration was practically the same between frozen–thawed and fresh hippocampal tissues (Kawato et al., 2025c). A possible reason of the discrepancy in nE2 concentration could be due to oxidation of 3-OH residue of nE2 by reactive oxygen species produced from mitochondria after losing of antioxidants and antioxidant enzymes by braking of cell membranes due to freezing of intercellular water and thawing of intercellular ices (Kawato et al., 2025c). On the other hand 3-O residue of nT is stable against oxidation stress. Therefore, fresh hippocampal tissues should be used for nE2 determination.

Interestingly, through electrophysiological methods, nE2 synthesis in male was indicated clearly. Rapid hippocampal E2 synthesis in adult male rats was revealed by LTP measurements of acute hippocampal slices (Grassi et al., 2011). Perfusion of letrozole (100 nM), an inhibitor of P450arom, in male hippocampal slices for 20 min considerably suppressed tetanus-induced LTP in CA1 region. These experiments clearly demonstrated the occurrence of such a rapid E2 synthesis.

It is also found that the perfusion of ICI 182,780 (ERα/ERβ blocker) (100 nM) in male rat hippocampal slices for 20 min considerably suppressed LTP in CA1, implying the involvement of synaptic membrane ERα as a receptor in this nongenomic signaling (Grassi et al., 2011; Hasegawa et al., 2015).

The local synthesis of nE2 in female mice hippocampus was also shown. In the case of hippocampal slices from OVX female mice and wild (not castrated) male mice, a significant impairment of CA1-LTP was observed one day after intraperitoneal injection with letrozole (10 μg/g body weight) (Vierk et al., 2012). The EPSP slope reduced to ~55% level in OVX female hippocampal slices and to ~80% level in male hippocampal slices. These letrozole treatments induced an impairment of LTP that is weaker in male mice than in female mice. It was explained in this paper that this weak LTP inhibition effect was observed due to a very low level of nE2 in male, and that this strong LTP inhibition was observed due to a high level of nE2 in female (Vierk et al., 2012). Another explanation for these results, however, might be possible, because wild male mouse hippocampus may have a higher nE2 concentration than OVX female as shown in several publications (Hojo et al., 2004; Kato et al., 2013), then letrozole-induced impairment effects on LTP could be weaker in male mice than OVX female mice.

Synthesis of nE2 was also indicated in the hippocampus of forebrain-neuron-specific aromatase knock-out (FBN-ARO-KO) mice by comparison with intact wild mice (Lu et al., 2019). For both male and female mice, a significant decrease in nE2 level was observed in the hippocampus of FBN-ARO-KO mice (Lu et al., 2019).

In summary, these investigations support a significant nE2 synthesis in adult male and female hippocampus, and might suggest a higher nE2 level in male hippocampus than in female hippocampus, not only in the early postnatal period, in which nE2 is necessary for male brain masculinization, but also in adult stage.

### 2.2 Comparison of nT and nDHT concentrations between male and female adult rats

Local synthesis of neuro-androgens (nT and nDHT) in male has been extensively studied, and reported concentrations of nT do not have serious disagreement between many publications (Hojo et al., 2009; Caruso et al., 2013; Hojo and Kawato, 2018; Li and Gibbs, 2019; Kawato et al., 2025c).

On the other hand, not much is known about local androgen synthesis in the female hippocampus. The accurate concentration of nT and nDHT in fresh (not frozen–thawed) female rat hippocampus was determined by using of LC–MS/MS with picolinoyl-derivatization of androgens (Supplementary Figure S1) (Hojo et al., 2014). The levels of nT and nDHT at Proestrus stage were ~ 1 nM and ~0.6 nM, respectively, suggesting a significant synthesis of nT and nDHT (Hojo et al., 2014). The concentration of plasma T was very low (~0.1 nM), implying almost no contribution of plasma T to hippocampal nT level. Metabolism analysis with tritiated T in hippocampal slices demonstrated the conversion of T to DHT, providing the direct evidence of 5α-reductase (DHT synthase) activity also in the female hippocampus.

In addition to local synthesis of nT, penetration of peripherally-derived T (cir-T) into the hippocampus could cause significant difference in nT concentration between male and female. Penetration of cir-T (~7 nM) into the male rat hippocampus might significantly contribute to keep the high level of nT (~17 nM) of male hippocampus (Figure 1B). On the other hand, in female rats (Figure 1B), penetration of circulating low level T (~0.1 nM at Proestrus stage) cannot contribute to the hippocampal nT level (~1 nM). Note that the level of circulating DHT was much lower than nDHT in both sexes, implying that nDHT was mainly synthesized from nT within the hippocampus.

Importantly, mRNA expression level of 5α-reductase (DHT synthase) was not significantly different across the estrous cycle, nor between female and male (Supplementary Figure S2). Steroidogenic enzymes (e.g., P450(17α), P450arom, StAR, P450scc, 3β-HSD) in female hippocampus were localized in CA1, CA3 and DG glutamatergic neurons as shown by immunohistochemistry (Supplementary Figure S3) (Hojo et al., 2014). Nearly no sex difference in the localization/expression patterns of steroidogenic enzymes was also observed in immunohistochemical pattern (Shibuya et al., 2003; Hojo et al., 2004; Hojo et al., 2014). Nevertheless, considerable sex differences exist with respect to the levels of nT, nDHT and nE2 between male and female hippocampus. One probable possibility is that these sex differences may be caused by the differences in the concentration of precursor steroids supplied from circulation, including cir-T and cir-PROG that penetrate into the hippocampus (see Figure 1B). Note that cir-T level is much higher in male than female, and in contrast, cir-PROG is much higher in female than male (Figure 1B).

The level of nAllo was determined at Proestrus stage as an indicator of 5α-reductase (Allo synthase) activity in female (Supplementary Figure S1). In contrast to androgens, the level of nAllo in female hippocampus was ~16 nM which was 16-fold higher than that in male. Note that the level of circulating Allo in plasma (<0.3 nM) was much lower than nAllo level.

## 3 Modulation effects of nE2, nDHT, and nT on LTP, LTD and spinogenesis

Synaptic plasticity is rapidly and nongenomically regulated by nE2, nDHT and nT, in addition to slow genomic regulation. We discuss LTP/LTD and spinogenesis, since they are two important aspects of synaptic plasticity.

### 3.1 LTP and LTD

#### 3.1.1 Excitatory effects of locally synthesized nE2 on LTP

The investigations of LTP (e.g., significant elevation of excitatory postsynaptic potential (EPSP) slope) have often been used for analysis of E2 effects on synaptic plasticity of hippocampal slices. Through extensive investigations over several decades, exogenously applied E2 has been shown to rapidly modulate LTP of CA1 synapses, as measured with EPSP or excitatory postsynaptic current (EPSC).

**Figure 2:**
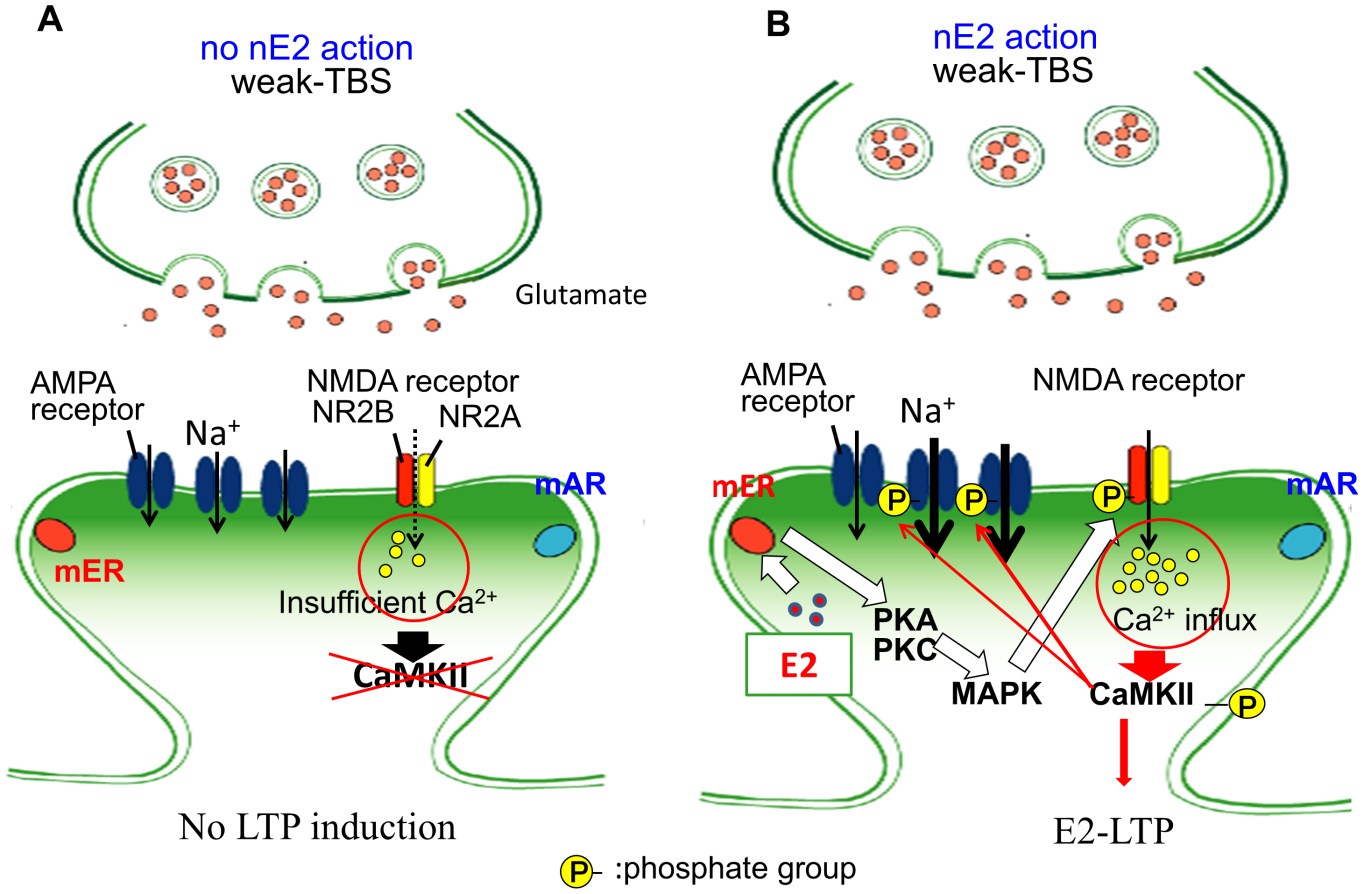
Schematic illustration for non-genomic modulation of E2 dependent LTP induction in rat hippocampus. LTP-induction in the presence and absence of E2 under weak (subthreshold) theta burst stimulation. **(A)** In the absence of E2, LTP cannot be induced. **(B)** In the presence of E2, the following signal cascade works: E2 drives kinases via mERα → kinase activation → phosphorylation NR2B subunit of NMDA receptor → increased Ca^2+^ influx → activation of CaMKII → phosphorylation of AMPA receptor → induction of LTP (E2-LTP) (modified from Hasegawa et al., 2015). It should be noted that DHT promotes mAR-mediated suppression of E2-LTP via activation of calcineurin (Hasegawa et al., 2015; Tozzi et al., 2019). mAR and mER are anchored to the membrane via palmitoylation. In female rat hippocampus, mERα forms complex with mGluR1, but not in male rat. It is not clear whether the involvement of ERα or ERβ in signaling pathway of LTP-induction is the same or not between mice and rats.

The increase of EPSP under subthreshold theta-burst stimulation was observed after 20 min perfusion of 10 nM E2 to in slices from 3 month-old male rats (Figure 2; Hasegawa et al., 2015).

However, in order to find the function of locally synthesized nE2, we should apply an inhibitor of nE2 synthesis, instead of application of exogenous E2.

Significant modulation effects of locally synthesized nE2 have been revealed by using letrozole (P450arom inhibitor). Brief application of letrozole induced the impairment of tetanus-induced LTP in rat hippocampal slices that are prepared from one month-old male rats (Grassi et al., 2011). Only 20 min perfusion of 100 nM letrozole considerably suppressed LTP in CA1 region of isolated hippocampal slices of adult male rats (Grassi et al., 2011).

On the other hand, in the case of OVX female mice and intact male mice, after *in vivo* intraperitoneal (i.p.) injection with letrozole (10 μg/g body weight), a significant impairment of theta-burst-induced CA1-LTP was observed in hippocampal slices (Vierk et al., 2012). One day after letrozole injection (i.p.), the EPSP slope reduced to ~55% in OVX female and to ~80% in male hippocampal slices, indicating sex differences (Vierk et al., 2012). Using genetically engineered mice (one month-old) which express only membrane ERα/ERβ but lacking nuclear ERα/ERβ, the interesting sex differences were observed in the magnitude of impairment on theta-burst-induced CA1-LTP in hippocampal slices (Wang et al., 2018). The impairment effects on LTP by the presence of formestane (P450arom inhibitor) was much greater in female hippocampus than in male (Wang et al., 2018). Concerning membrane ERα/ERβ participation in LTP induction, only membrane ERα but not membrane ERβ was participated in genetically engineered female mice. In male, however, only membrane ERβ but not membrane ERα was participated in LTP induction (Wang et al., 2018).

FBN-ARO-KO mice are also useful to investigate the function of nE2, since nE2 production is absent in the hippocampus (Lu et al., 2019). In both male and OVX female FBN-ARO-KO mice, tetanus-induced CA1-LTP in hippocampal slices was significantly impaired as compared with wild intact mice (Lu et al., 2019). Acute 1 nM E2 administration rescued the impaired LTP in these all FBN-ARO-KO mice. These results suggested that sex differences were small and that nE2 in both male and female FBN-ARO-KO mice is necessary for LTP-induction.

#### 3.1.2 Effects of nE2 and nDHT are differently appeared on LTP and LTD

Effects of nE2 are excitatory on LTP but effects of nDHT are inhibitory on LTP. It is shown that a short-term E2 perfusion (10 nM E2) induced LTP in CA1 of adult male rat hippocampus (3 month-old rat) under subthreshold theta burst stimulation (Figure 2; Hasegawa et al., 2015). Figure 2 illustrates nE2-dependent nongenomic signal cascade through “mERα → kinase activation (PKA, PKC, MAPK) → phosphorylation of NMDA receptor → increase in Ca^2+^ influx → activation of CaMKII → phosphorylation of AMPA receptor → induction of LTP (E2-LTP).”

Interestingly, this E2-dependent LTP was impaired by co-perfusion of DHT with E2 when DHT level was more than 3-fold of E2 level (Hasegawa et al., 2015). Single cell patch-clamp analysis revealed that locally produced nDHT is involved in the induction of LTD with low-frequency stimulation in male rat CA1 hippocampus (Di Mauro et al., 2015; Tozzi et al., 2019). The AR blocking with flutamide prevented LTD. Within the same cell, nE2 dependent LTP was shown, as observed by LTP impairment by blocking of ER with ICI 182,780 and also by blocking of nE2 synthesis with letrozole. Therefore, both nE2-dependent LTP and nDHT-dependent LTD can be expressed in the same pyramidal neurons. These results suggest that nDHT might show suppressive effects on synapses when E2 induces hyper-excitability of synapses.

### 3.2 Regulation of dendritic spines and LTP by nE2, nDHT, and nT: depending on protein kinase networks and membrane receptors

#### 3.2.1 Both nE2 and nDHT non-genomically increase dendritic spines

**Figure 3:**
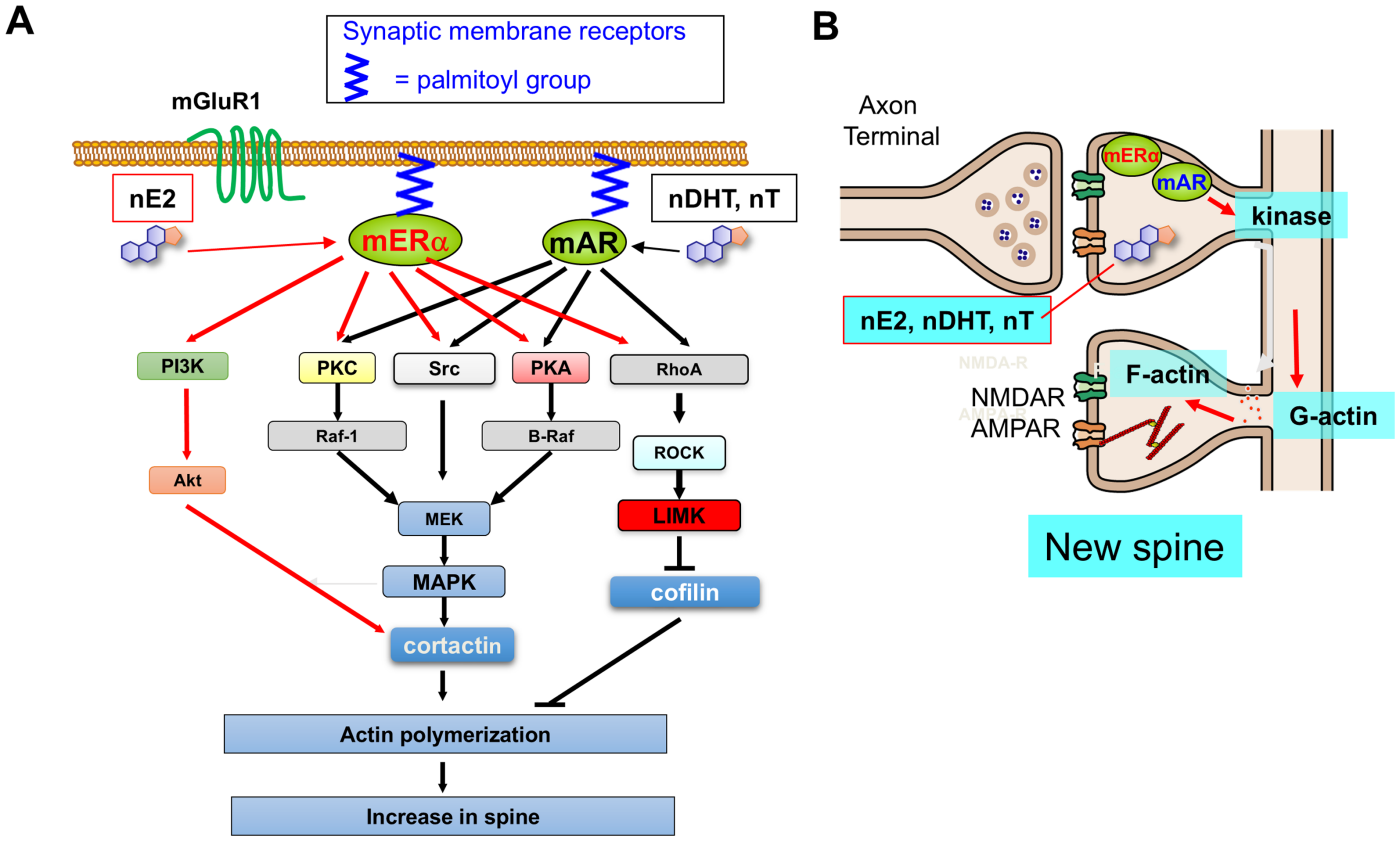
**(A,B)** Schematic illustration for non-genomic modulation of dendritic spine formation induced by nE2, nDHT, and nT in rat hippocampus. Upon binding of nE2 to mERα, the following signal cascade works: nE2 drives several kinases (PKA, PKC, MAPK, LIMK, and Src) → phosphorylation of cortactin and cofilin → actin polymerization → new spine formation. Upon binding of DHT and T to mAR, the same signal cascades occur resulting in new spine formation. mERα and mAR are anchored to the membrane via palmitoylation. In male, mERα does not form complex with mGluR1, but in female mERα forms complex with mGluR1 (Boulware and Mermelstein, 2009) (modified from Hasegawa et al., 2015; Hatanaka et al., 2015; Soma et al., 2018).

The rapid nongenomic modulatory actions of E2 and DHT on dendritic spines have been extensively studied for neurons in hippocampal CA1 region. Here recently, fluorescently labeled dendritic spines were analyzed with 3-dimentional confocal imaging, in addition to analysis of 2 dimensional images with Golgi staining. Very interestingly, not only E2 but also DHT (at 1 ~ 10 nM concentration) increased the spine density within 2 h in hippocampal CA1 region of isolated male slices (Figure 3) (Mukai et al., 2007; Hasegawa et al., 2015; Hatanaka et al., 2015). Surprisingly, this DHT-induced spine increase in male rats is clearly different from DHT-induced LTP regulation mechanisms in which DHT showed suppressive effects on E2-supported LTP, as described in Section 3.2.1 (Hasegawa et al., 2015; Di Mauro et al., 2015; Di Mauro et al., 2017; Tozzi et al., 2019).

Not only E2 perfusion in isolated hippocampal slices but also *in vivo* E2 infusion into the brain of living mice are used for investigations. *In vivo* infusion of E2 into the dorsal hippocampus of OVX female mice increased the spine density rapidly within 0.5 ~ 2 h (Luine and Frankfurt, 2012; Jacome et al., 2016; Tuscher et al., 2016a).

Electron microscopic analysis of spine-synapses demonstrated that the density of CA1 hippocampal spine-synapse was regulated by androgen and estrogen. The density of CA1 spine-synapses was increased upon subcutaneous (s.c.) injection of DHT and T (1.6 mg/kg body) for 3 days in castrated male rats in which the spine-synapse density was already decreased by castration (Leranth et al., 2003). It was found that the rapid E2-induced increase (within 30 min) of the CA1 spine-synapse density occurred in OVX female rats upon s.c. injection of relatively high dose E2 (45 μg E2/kg body) (MacLusky et al., 2005; Kuwahara et al., 2021). However, such an increase in the spine-synapse density did not occur upon injection of low dose E2 (15 μg E2/kg body).

#### 3.2.2 Kinase signaling in rapid nongenomic modulation of spines and LTP by nE2 and nDHT

In order to analyze the mechanisms of rapid nongenomic modulation of spines and LTP, not only membrane receptors of nE2 and neuro-androgen but also downstream kinase-dependent signaling have been investigated (Hasegawa et al., 2015; Hatanaka et al., 2015; Tuscher et al., 2016a; Wang et al., 2018; Jain et al., 2019; Lu et al., 2019; Sheppard et al., 2023).

First, we describe kinase-dependent signaling in this Section 3.2.2, and we next describe membrane estrogen/androgen receptors that are responsible for both spinogenesis and LTP in Section 3.2.3.

As a candidate for downstream signaling of membrane receptors of nE2 and nDHT, kinase-dependent signaling has been examined for E2- and DHT-induced rapid nongenomic increase of spine density in isolated male rat hippocampal slices. Co-treatments of kinase inhibitors with E2, T or DHT are used to find a target kinase. Interestingly, with co-treatments of E2 with inhibitors of Erk MAPK, PKA, PKC, LIMK or Src kinase, E2-induced spine increase was considerably suppressed (Hasegawa et al., 2015; Soma et al., 2018). Also, with co-treatments of DHT or T with inhibitors of Erk MAPK, PKA, PKC, LIMK, and Src kinase, DHT-induced spine increase and T-induced spine increase were considerably suppressed (Hatanaka et al., 2015; Soma et al., 2018). Mechanisms of spine density regulation can be explained as follows: binding of E2 and DHT to mER and mAR → activation of kinases →phosphorylation of cortactin and cofilin→ actin polymerization → spine increase (Figure 3).

In case of OVX female mice dorsal hippocampus *in vivo*, involvement of MAPK and PI3K is indicated from the observation that rapid E2-induced spine increase was suppressed by *in vivo* co-infusion with kinase inhibitors (Tuscher et al., 2016a; Sheppard et al., 2023).

Kinase-dependent signaling has also been investigated electrophysiologically observing EPSP-LTP and EPSC (with voltage clump methods) in isolated rat hippocampal slices. As already described in Section 3.2.2, in male, involvement of PKA, PKC and MAPK (but not Src kinase) was indicated in EPSP-LTP using combination of short-term E2 (10 nM) perfusion and subthreshold theta burst stimulation (Figure 2) (Hasegawa et al., 2015).

Involvement of MAPK signaling in tetanus-induced-LTP was also found in FBN-ARO-KO mice (male and female) upon acute E2 (1 nM) perfusion with and without MAPK inhibitor (Lu et al., 2019). On the other hand, involvement of PKA, MAPK and Src kinase was observed in female by observing changes of EPSC upon acute nE2 (100 nM, extremely high concentration) supplementation with and without kinase inhibitors (Jain et al., 2019). Interestingly, MAPK and Src kinase were also involved in male signaling, but PKA was not involved in male.

Taken together, it should be noted that different kinases are involved in different types of nongenomic signaling.

#### 3.2.3 Membrane receptors of nE2, nT, and nDHT in nongenomic function of LTP and spinogenesis

Rapid nongenomic actions of nE2 and nDHT require membrane receptors, because these events are membrane-initiated ones (Levin and Hammes, 2016; Mauvais-Jarvis et al., 2022). Hippocampal expression of nuclear estrogen and androgen receptors, including ERα, ERβ and AR, has been indicated in principal neurons (e.g., glutamatergic neurons) with immunostaining and immunoelectron-microscopic investigations (Milner et al., 2005; Tabori et al., 2005; Mukai et al., 2007). Since nuclear estrogen and androgen receptors, such as nuclear ERα (nERα), nuclear ERβ (nERβ) and nuclear AR (nAR), are not fitted to the mechanisms of rapid effects, synaptic membrane receptors (extra-nuclear receptors) for estrogen and androgen have been searched as candidates by which rapid effects are mediated. Because blockers for nER and nAR were also very effective to prevent rapid effects of E2 and DHT on modulation of LTP and spinogenesis, membrane bound receptors, such as membrane ERα (mERα), membrane ERβ (mERβ) and membrane AR (mAR), are thought to be promising candidates (Hasegawa et al., 2015; Hatanaka et al., 2015). However, the question of how mER and mAR are localized at synaptic membranes has been complex and still unsolved. A candidate of membrane binding form of mER and mAR may be palmitoylated form (Figure 3), as deduced from many investigations of Levin’s group (Pedram et al., 2014; Levin and Hammes, 2016), Fukata’s group (Fukata and Fukata, 2010) and Mermelstein’s group (Meitzen et al., 2013; Meitzen et al., 2019).

Interestingly, concerning mER-initiated signaling, not only sex-differences but also animal differences between rats and mice are observed in several cases.

Many publications support that in male rats only ERα but not ERβ was participated in E2-induced rapid nongenomic spinogenesis (Mukai et al., 2007).

In OVX female mice, only mERα but not mERβ was participated in E2-induced rapid nongenomic spine increase in CA1 was observed, in addition, E2-induced rapid nongenomic behavioral improvement was also found, including object recognition and object placement (Phan et al., 2011; Phan et al., 2015; Paletta et al., 2018).

However, in another OVX female mice study, only mERβ but not ERα was observed to participate in LTP and spinogenesis (Liu et al., 2008). In genetically engineered male mice, only mERβ but not mERα was participated in LTP induction (Wang et al., 2018). As such, at the present time, we still do not have solid agreement about selective involvement of mERα and mERβ in nongenomic modulation of synaptic plasticity.

As another type of membrane-initiated signaling, complex formation of palmitoylated mERα/mERβ with mGluR1 through caveolin was indicated to play an important role in membrane-initiated E2 signals of female rat hippocampus but not male (Boulware and Mermelstein, 2009; Meitzen et al., 2013; Meitzen et al., 2019). Binding of E2 to mERα/mGluR1 complex induced CREB phosphorylation through Erk MAPK in female hippocampal primary cultured neurons, but not in male neurons (Boulware and Mermelstein, 2009). In male rat hippocampus mERα/mGluR1 complex formation was not involved in LTP-induction and spinogenesis (Hasegawa et al., 2015). Investigations of E2/DHT/T signaling through complex formation of mERα/mERβ/mAR with mGluR1/mGluR2/mGluR5 in male and female are very useful to find clear sex-differences in the hippocampal synaptic plasticity.

## 4 Rapid effects of nE2 and nT on cognitive behavior. OVX- and castration-induced impairment of cognitive behavior and E2- and T-induced rescue

A significant progress has been achieved about nE2 effects on cognitive behavior in female rodents. Importantly, depending on estrous cycle, intact female rats have nE2 level of ~1 nM (Estrus and Diestrus stages) and ~4 nM (Proestrus stage) in the hippocampus, and OVX decreased nE2 down to ~1 nM (Kato et al., 2013). Therefore, OVX can cause impairment of cognitive performance and E2 infusion can be effective to rescue cognitive impairment (Tuscher et al., 2016b). In OVX female mice, rapid changes occurred in cognitive behaviors including object recognition and object replacement within 1 h after E2 infusion into the dorsal hippocampus (Tuscher et al., 2016b; Kim and Frick, 2017; Taxier et al., 2020; Luine and Frankfurt, 2020; Sheppard et al., 2023).

These cognitive impairments in OVX female mice are rapidly rescued by E2 replacement through MAPK-dependent and PI3K-dependent modulation of synapse formation (Tuscher et al., 2016a; Sheppard et al., 2023). Also investigations of OVX female rats showed that E2 replacement induced rapid activation of cognitive behavior (object replacement) (2–4 h) and increase in spines (1 h) (Jacome et al., 2016; Luine and Frankfurt, 2020).

Importance of local synthesis of nE2 is also indicated from cognitive impairments of intact male mice induced by letrozole infusion into the mouse dorsal-hippocampus.

In intact/wild male mice, infusion of letrozole (ER blocker) only and flutamide (AR blocker) only into the dorsal hippocampus did not impair object placement and object recognition tasks (Koss and Frick, 2019), indicating that both ER and AR signals actively work together in intact males. In fact, co-administration of both letrozole and flutamide to intact male mice successfully blocked memory consolidation of these two tasks (Koss and Frick, 2019). In castrated male mice, on the other hand, infusion with letrozole only into the hippocampus did impair object placement and object recognition tasks (Koss and Frick, 2019). These results indicate that combination of castration (depletion of peripherally-derived T) and letrozole infusion (inactivation of local conversion of nT to nE2) can successfully impair cognitive performance.

These results may be in good agreement with the reports that castration did not decrease nE2 (as high as ~8 nM) in male rat hippocampus (Hojo et al., 2009; Hojo and Kawato, 2018; Kawato et al., 2025c). Therefore, letrozole-induced nE2 depletion is necessary for cognitive impairment of male, in addition to considerable decrease in nT by castration (for example, from ~17 nM to ~3 nM in male rat hippocampus) (Hojo et al., 2009). Importantly, these investigations indicate that intact/wild male rats locally synthesize nE2 in the hippocampus.

Even in the case of female, an essential role of androgen on cognition was demonstrated in rats. Letrozole only did not alter recognition memory in OVX female rats and did not block the effects of testosterone propinate (TP) supplementation on recognition memory (Luine et al., 2022). TP improved recognition memory and increased spine density in OVX female rats (Luine et al., 2022). These results suggest that memory improvement effects are mediated via both nE2 and nT.

FBN-ARO-KO male and female mice (together with intact male, intact female and OVX female) were used to investigate nE2 depletion effects on hippocampal-dependent spatial reference memory and recognition memory, by using Barnes Maze test and novel object recognition test (Lu et al., 2019). Aromatase-KO exhibited significant deficits in these spatial reference memory and recognition memory. Surprisingly, Aromatase-KO-induced impairments were observed in Barnes Maze test and novel object recognition test in comparison with other three reference mice (including intact male, intact female and OVX female), implying the necessity of nE2 for cognition for not only female but also male mice (Lu et al., 2019).

In addition, effects of T on spatial memory using castrated adult male rats were investigated with behavioral test in combination with TP-supplementation (Wagner et al., 2018). In order to improve spatial memory, castrated male rats were treated with TP by s.c. injection for 7 days, and were undergone a radial-arm maze test. Dose response of TP supplementation was complex. T-induced improvement was observed at T dose with 0.125 mg and 0.5 mg, but not 0.25 mg and 1.0 mg. TP supplementation can elevate nT concentration, but nE2 concentration might not be changed, as suggested from the following reason. Castration of male rats can considerably decrease nT (down to 20–30%) in rat hippocampus, but castration cannot significantly decrease nE2 concentration (Hojo et al., 2009). Therefore, castration-induced deprivation of circulating T only may not be able to significantly impair the local nE2 action, since male rat hippocampus has both androgen and estrogen signaling pathways that maintains the spine density (Hasegawa et al., 2015; Hatanaka et al., 2015; Koss and Frick, 2019), Therefore, T supplementation only might not be able to sufficiently improve spatial memory performance of castrated male rats. Note that, castration should be performed by an expert of animal surgery, because castration operation by a non-expert person (including PhD students) might decrease the spine density due to artificial micro-ischemia during operation (Kawato et al., 2025c), leading to decrease in behavior test score.

## 5 Aging-induced cognitive decline in relation to decrease of nT and nE2

### 5.1 Aging-induced neuronal vulnerability and cognitive decline

During the normal aging process, animals experience age-related cognitive decline. Historically, it was thought that primary contributions to the aetiology of this decline were massive cell loss (Brody, 1955) and deterioration of dendritic branching (Scheibel et al., 1976). However, recently we know that the changes occurring during normal aging are more subtle and selective than was once believed. In fact, the general pattern seems to be that most age-associated behavioral impairments result from region-specific changes in dendritic morphology and synaptic transmission which support cognition.

Of the brain regions affected by aging, the hippocampus and the prefrontal cortex (PFC) seem to be particularly vulnerable, but even within and between these regions the impact of aging on neuronal function differs.

In case of normal aging, the number of neurons in the hippocampus and PFC do not change along with aging (Morrison and Hof, 1997; Morrison and Hof, 2002; Rapp and Gallagher, 1996; Rasmussen et al., 1996; West et al., 2004; Burke and Barnes, 2006; Kelly et al., 2006). This is very different from the case of aging-dependent pathological cognitive decline such as Alzheimer’s disease (AD) that shows neuronal cell death or cell loss (West et al., 1994; Morrison and Hof, 1997; Morrison and Hof, 2002).

These results suggest that the decline in learning and memory along normal aging is likely to occur at a synaptic level, because of no neuron loss. In fact, the general pattern seems to be that most age-associated behavioral impairments result from region-specific changes in dendritic morphology and neural connectivity, Ca^2+^ dysregulation (Thibault and Landfield, 1996) that affect plasticity and ultimately alter the network dynamics of neural ensembles that support cognition.

Aged rats have deficits in both LTP induction and maintenance (Burke and Barnes, 2006). There is a reduction in the field EPSPs recorded both in the hippocampal CA1 (Barnes et al., 1992; Deupree et al., 1993) and hippocampal dentate gyrus (Foster et al., 1991), and aged rat neurons in area CA1 show LTP induction deficits, when peri-threshold stimulation parameters were used (Barnes et al., 1996; Deupree et al., 1991; Moore et al., 1993).

Along normal aging, a significant decrease of CA1 spine density occurs in female Fischer 344 rats (Luine et al., 2011) and in male Wistar rats (Kawato et al., 2025a). No changes in CA1 spine density was, however, reported in Long Evans rats between aged and young rats (Markham et al., 2005). Differences in animal and strain might affect region specific vulnerability in these results.

Since the hippocampus and the PFC are particularly vulnerable to the aging process, performance on tasks that require information processing in these regions declines with age (Rosenzweig and Barnes, 2003). One such example is an age-related decline in spatial memory. Deficits on spatial navigation tasks are observed in a variety of aged animals, including aged rats (Barnes, 1979; Markowska et al., 1989; Gallagher and Rapp, 1997), mice (Bach et al., 1999), monkeys (Lai et al., 1995; Rapp et al., 1997), and humans (Wilkniss et al., 1997).

### 5.2 Aging-dependent male cognitive decline depending on decrease of T and E2

Many researches have been conducted to find the possibility that the aging-dependent decline in cognition and neural activity (evaluated by LTP and spine density described in Section 5.1) is induced by a decrease in neurotrophic factors in the hippocampus and PFC. Neurotrophic factors include neuro-androgen (nT and nDHT), neuro-estrogen (nE2), brain-derived neurotrophic factor (BDNF) and nerve growth factor (NGF). It is very important, however, that there was no age-dependent decrease of BDNF and NGF levels along normal aging of the hippocampus but their levels remain almost constant (Perovic et al., 2013; Kawato et al., 2025a).

Therefore, this Section 5.2 describes the researches aiming to elucidate the neural activity decline associated with cognitive decline caused by aging in male rodents and humans, which is accompanied by a decrease in T and E2 levels in males at the neuron level. Because the synaptic effect of E2 in male is as significant as that of T and DHT, not only T but also E2 should be investigated in the male brain including the hippocampus.

**Figure 4:**
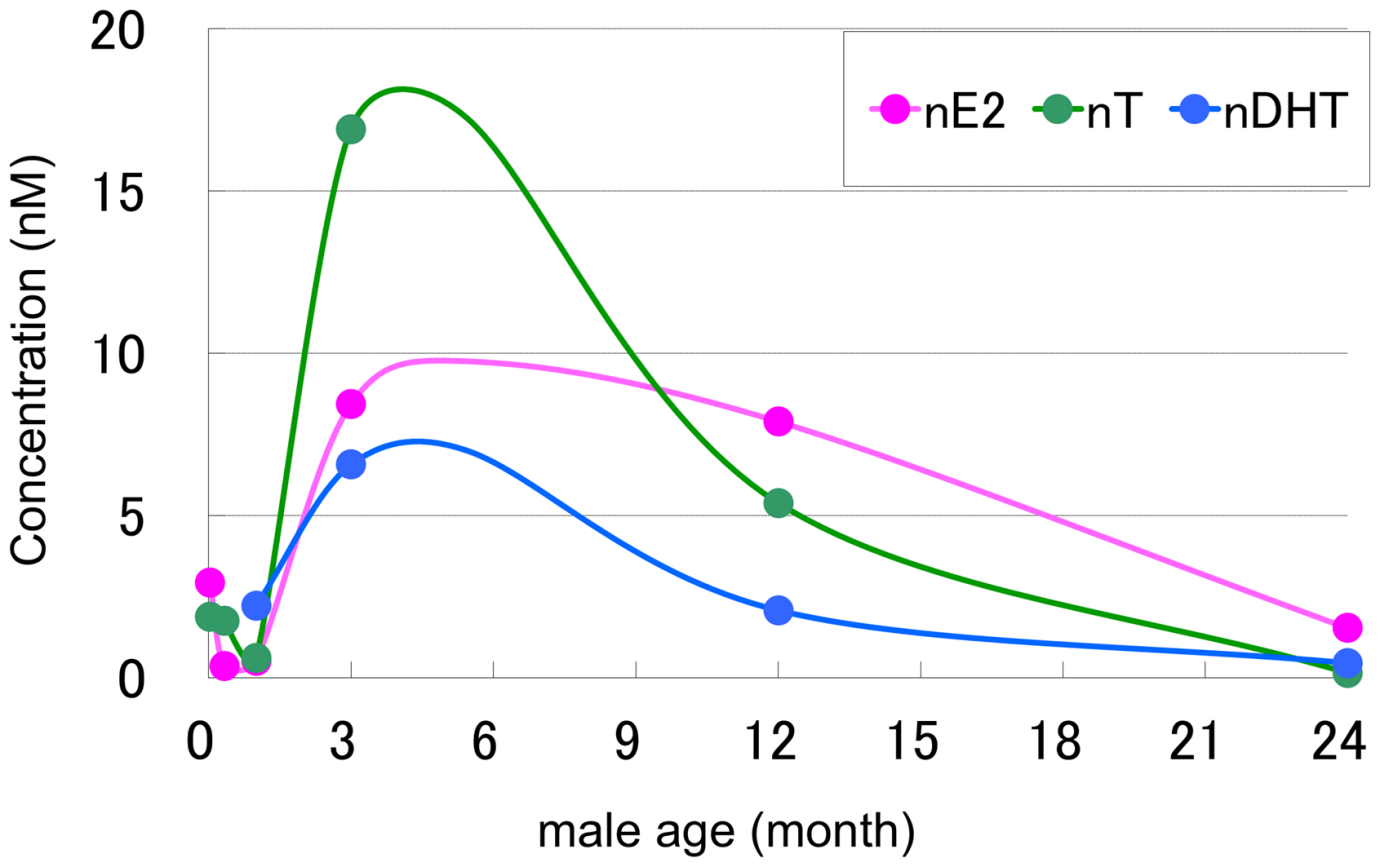
Age-induced decrease in nE2 (pink), nT (green), and nDHT (blue) concentrations in the hippocampus from male rats at 3 month-old (3 m), 12 month-old (12 m), and 24 month-old (24 m). Note that circulating cir-E2, cir-T, and cir-DHT are much lower than nE2, nT, and nDHT (see Figure 1). Additionally, nE2, nT, and nDHT in postnatal 1, postnatal 10, and 4 weeks rat hippocampus are shown together. The concentration of nE2, nT, and nDHT are analyzed in freshly prepared hippocampus (not frozen–thawed hippocampus) by using LC–MS/MS (modified from Hojo et al., 2009; Kawato et al., 2025a). Note that the circulating T level in 24 m is much lower than nT (Kawato et al., 2025a). Plasma T considerably decrease from ~11 nM (3 m) to ~0.3 nM (24 m), and plasma DHT are the same around ~0.5 nM between 3 m and 24 m. Male plasma E2 level is very low around ~0.02 nM between 3 m and 24 m (Kawato et al., 2025a). Note that the female concentration of nE2 at 3 m [~4 nM (Proestrus) and ~1 nM (estrus, diestrus)] is much lower than that of male nE2 (~ 8 nM) (Kato et al., 2013).

The concentrations of nT, nDHT and nE2 in the (freshly prepared) male rat hippocampus showed clear age-dependent decrease from [~17 nM (nT), ~7 nM (nDHT) and ~8 nM (nE2)] in 3-month-old (3 m) to [~0.2 nM (nT), ~0.4 nM (nDHT) and ~2 nM (nE2)] in 24-month-old (24 m) (Kawato et al., 2025a; Kawato et al., 2025c). Notably, the concentration of nT and nDHT decreased greatly to nearly 1/100 level by going from 3 m to 24 m, resulting in loosing functions as synaptic modulators (see Figure 4). On the other hand, nE2 concentration decreased moderately (and not greatly) to nearly 1/4 level by going from 3 m to 24 m. Therefore, in 24 m, nE2 is a dominant neurotrophic factor, controlling synaptic plasticity of male hippocampus.

Age-dependent decline of synaptic plasticity was also investigated with the CA1 spine density of male rats (Kawato et al., 2025a). A moderate decrease of CA1 spine density was observed from ~2.3 spines/μm (3 m) to ~1.7 spines/μm (24 m), which corresponds to approx. 26% decrease. Moderately high level of nE2 (~2 nM) probably prevents further drastic decrease in the spine density, although nT and nDHT are extremely low (<0.5 nM) in 24 m hippocampus. A significant recovery of the spine density of CA1 hippocampus with a single androgen application in 24 m was observed. Ten hours after a single supplementation of DHT and T (1 mg/kg) increased the spine density by nearly 8%, showing a good responsiveness to neuro-androgen.

Concerning the synthesis of androgen, age-dependent decline occurred in androgen synthesis enzymes and androgen receptor as observed by mRNA expression levels in male rats (Kawato et al., 2025a). The expression levels of androgen synthesis enzymes, including DHEA synthase [P450(17α)], testosterone synthase [17β-hydroxysteroid dehydrogenase (17β-HSD)], and DHT synthase [5α-reductase] have been investigated.

The level of these mRNAs significantly decreased from 100% (3 m) to approx. 60–75% (24 m), except for no decrease in 5α-reductase 1. AR mRNA showed age-induced decrease from 100% (3 m) to 85% (24 m). Taken together, it is suggested that androgen signaling pathway is decreased along normal aging.

In contrast, mRNA of estrogen synthesis enzyme P450arom and estrogen receptor ERα did not change with age (Kawato et al., 2025a). It is therefore suggested that E2 signaling pathway does not significantly decline along aging. Note that, however, ERβ mRNA decreased to ~60% (24 m).

On the other hand, importantly, the contribution of circulating T, DHT and E2 to hippocampal neural plasticity would be very small. The concentrations of circulating T, DHT and E2 in 3 m and 24 m male hippocampus are always much lower than those of hippocampal nT, nDHT and nE2 (Figure 4). In circulation, plasma T considerably decreased from ~11 nM (3 m) to ~0.3 nM (24 m) and plasma DHT did not further decrease, with being the same level around ~0.5 nM (Kawato et al., 2025a). The circulating T level in 24 m were extremely lower than that in 3 m. Male plasma E2 levels did not change, with being the same low level around ~0.02 nM during 3 m, 12 m and 24 m. In these comparisons, note that plasma sex-steroid concentrations are about a double of blood concentrations due to their volume difference between plasma and whole blood.

Historically, many investigations, including administration, replacement, and subtraction of androgens, have been performed in order to reveal the mechanisms of androgen effects on synaptic plasticity of the brain. For example, perfusion of T and DHT significantly increased the dendritic spine density within 2 h in young adult rat hippocampal slices (Hatanaka et al., 2015). These rapid spine increases were driven via membrane synaptic AR receptor and kinase signaling. T administration increases synaptic density in the hippocampal dentate gyrus of old mice (Fattoretti et al., 2019). After castration of young male rats in order to decrease serum T, T replacement improved spatial memory (Wagner et al., 2018). T replacement also improved spatial memory in aged male rats in which serum T level was very low (Jaeger et al., 2020).

In male human, interestingly, the decrease in brain T level along aging of elderly men (50 to 97 years old) was observed (Rosario et al., 2004), in which brain T was purified from the mid frontal gyrus of neuropathologically normal men. From a comparison between neuropathologically normal men and AD men, AD men had lower brain T level than neuropathologically normal men (Rosario et al., 2004).

Male human data indicate a significant decline in circulating T due to chronological aging (Harman et al., 2001; Morley et al., 1997; Travison et al., 2007). In male human, age-dependent decrease in serum T level is closely associated with cognitive decline in addition to sarcopenia, depressed mood, and frailty (Travison et al., 2007).

Late-onset hypogonadism (LOH) in male human is defined as a disease characterized by age-related T decline and associated clinical symptoms. LOH may have a close relationship with andropause in male.

LOH patients exhibit symptoms that include hot flashes, depression, fatigue, and decreased libido due to decreased circulating total T and free T. The main symptoms of LOH are well evaluated by the Aging males’ symptoms scale (AMS), self-rating depression scale (SDS), and the international index of erectile function score (IIEF-5) (Heinemann et al., 2003; Lunenfeld et al., 2013; Ide, 2023; Ide et al., 2023).

T replacement therapy (TRT) has been widely applied to improve physical and mental impairment induced by LOH. TRT improves health-related QOL (quality of life) in patients with LOH (Borst et al., 2014; Corona et al., 2016; Corona et al., 2021; Ide, 2023).

Numerous investigations have shown a good relationship between T-loss and decline of muscle and physical ability (i.e., sarcopenia) (Gregori et al., 2021; Ide et al., 2023).

However, a clear evidence had not been shown on the matter indicating that T-loss along human aging declines cognitive functions (Borst et al., 2014; Corona et al., 2016; Corona et al., 2021). There are several cognitive abilities tested, including attention, memory, calculation, verbal ability, auditory and visual processing. However, many previous studies suggest that these general cognitive abilities did not show significant improvement in older men (60–80 years old) by long term TRT (for half a year or two years) (Borst et al., 2014; Lisco et al., 2020; Gregori et al., 2021).

We recently applied spatial navigation task test and successfully observed improvement effects of spatial cognitive function by T-replacement therapy (TRT) on male patients with LOH (Kawato et al., 2025b). LOH patients have significantly low levels of T as well as high AMS scores when compared with healthy men. Upon TRT for 6 weeks, it was revealed that the spatial cognitive ability of LOH patients was significantly improved along the increase of total T levels, free T levels and improvement of AMS scores (Kawato et al., 2025b).

This achievement can be explained by following several reasons. Male human is good at spatial navigation task. Using a virtual maze navigation game created on a PC to investigate spatial cognitive function, many reports indicated that men are significantly faster than women at finding the exit of the maze. The reported difference in spatial ability between men and women may be due to higher T levels in male than in female (Hojo et al., 2009; Ooishi et al., 2012; Kato et al., 2013). Male spatial navigation ability is hippocampus dependent, since men show significantly increased activity in the hippocampus during spatial navigation task as judged from functional MRI neuroimaging (Grön et al., 2000). The hippocampal CA1 region is known to be essentially responsible to spatial navigation ability, because the glutamatergic neurons in the CA1 region are responsible for spatial cognition as evidenced by the fact that spatial cognition is selectively inhibited in mice in which N-methyl-D-aspartate (NMDA) receptors of CA1 glutamatergic neurons are specifically knockout (Tonegawa et al., 1996; McHugh et al., 1996). In addition, AR receptors are localized in CA1 neurons (Tabori et al., 2005) and the application of T can increase CA1 spine density in rats (Hatanaka et al., 2015). Taken together, it can be concluded that TRT can increase hippocampal T level of LOH patients, leading to recover the spatial cognition ability of LOH patients. It should be noted that the LOH patients in the above TRT study was not older men (60–80 years old) but rather middle-aged men (between 45 and 60 years old) who have more neural plasticity than older men, and this may be one reason why the relatively short term TRT (6 weeks) could show effective improvements. In addition, subjects (LOH patients, middle-aged) did not show mild cognitive impairment (MCI) symptoms or dementia, by using Mini-Mental State Examination (MMSE) (Gong et al., 2021; Arevalo-Rodriguez et al., 2015), suggesting that subjects may have semi-healthy neural plasticity in the hippocampus.

In contrast to normal aging of male animals, pathological diseases, including AD and Senescence Accelerated Mouse-Prone 8 (SAMP8), cause cognitive decline including significant neural disorder and cell death. AD induces neurological disorders via formation of amyloid β plaques and neurofibrillary tangles due to tau-aggregation. In male AD model of 3xTg-AD triple transgenic male mice, androgen depletion significantly accelerated AD-like neuropathology. Supplementation of T and DHT in 3xTg mice considerably reduced amyloid β accumulation, indicating a significant inhibitory effects of androgen on AD development (Rosario et al., 2010).

Impairment of LTP in AD model male rats was investigated, using hippocampal slices treated with soluble amyloid β42 aggregates. LTP in dentate gyrus (DG) of intact slices was impaired by amyloid β treatments (Bellingacci et al., 2024). This DG-LTP impairment was blocked upon supplementation of finasteride (inhibitor of 5α-reductase), via increase of nE2 by inhibition of conversion from nT to nDHT. Note that the necessity of nE2 for LTP was directly indicated by letrozole-induced LTP impairment.

SAMP8 mouse is a senescence accelerated mouse which has been widely used for pathological aging model. SAMP8 exhibits cognitive impairment and hypogonadism. Investigations of male SAMP8 mouse showed the relationship between plasma T decline and age-related neural dysfunction (Ota et al., 2012). Interestingly, T and DHT replacement in male SAMP8 inhibited cognitive decline through an increase of sirtuin 1 protein (SIRT1) expression (Ota et al., 2012).

Taken together, androgen plays an important role in protection against cognitive decline depending on not only normal aging but also AD-related diseases and senescence-accelerating diseases.

### 5.3 Aging-induced female cognitive decline dependent on decrease of nE2 and circulating E2

In female, cognitive decline may be significantly influenced by a decrease in cir-E2 and nE2, during and after menopause which occurs due to dysfunction of Hypothalamus-Pituitary-Gonadal axis. Estrogen plays a neuroprotective role in the brain, therefore its decline can be associated with various cognitive changes, including memory issues. Note that circulating E2 concentration is approx. Half of plasma or serum E2 concentration (see Figure 2B).

In young adult rodents, many studies have shown that E2 supplementation enhances LTP and spines in hippocampal slices of both female and male rodents (Bi et al., 2000; Foy et al., 1999; Hasegawa et al., 2015; Mukai et al., 2007; Mukai et al., 2010; Ooishi et al., 2012; Vouimba et al., 2000). However, in normally aged rodents, these types of morphological and electrophysiological investigations upon E2 supplementation have not been sufficiently performed (Adams et al., 2001; Morrison et al., 2006).

During and after menopause, not only cir-E2 but also nE2 levels decrease, reaching probably to nearly the same level in OVX adult or diestrus adult animals (menopause model) (Kato et al., 2013). A possible decrease in the spine density after menopause could be estimated as follows. In young female rats, nE2 level was ~0.7 nM in Diestrus-2 stage with the spine density of ~2.0 spines/μm, on the other hand, nE2 at Proestrus was ~ 4 nM and the spine density was ~2.4 spines/μm (Kato et al., 2013). After menopause, the endocrine state may be close to diestrus-2 stage, then the spine density might be ~2.0 spines/μm, if we assume that the spine density is determined by only the nE2 concentration in female having low nT (Kato et al., 2013). As experimental results, along normal aging, the hippocampal CA1 spine density significantly decreased and spatial memory deficit occurred in female fischer 344 rats (21 month-old) (Luine et al., 2011).

In next step, E2 replacement effects on normally aged rat hippocampus are examined. E2 replacement effects in young OVX and aged animals have been investigated in order to examine methods of improving impairments caused by menopause. Interestingly and surprisingly, E2 replacement effects on hippocampal CA1 spines are very different between young and aged animals. Although E2-induced increase in CA1 spine density was observed in young OVX rats, such a spine density increase was not observed in normally aged rats (Adams et al., 2001). Age-related attenuation of the beneficial cognitive effects of estrogen manipulations in rats was also observed (Markowska and Savonenko, 2002).

Different from the situation of rats, in CA1 of both young and normally aged monkeys, E2 induced a significant increase in the total spines (Hao et al., 2003). Unlike female rat, aged female monkey retained the capacity for such an increase in spines, which is particularly relevant to women, given the similarities in patterns of endocrine senescence between humans and monkeys. The beneficial effects on dendritic spine increase by E2 replacement have been observed clearly in aged female rhesus monkey, particularly in PFC (Hao et al., 2006; Hao et al., 2007; Hara et al., 2015; Morrison et al., 2006; Morrison and Baxter, 2012).

In contrast to normal aging, AD is a serious pathological disease in female cognitive decline along aging. It is suggested that a probability of getting AD in women may be significantly higher than that in mem (Pike et al., 2009; Carroll et al., 2010).

As a rodent model of female AD, 3xTg-AD triple transgenic female mice were used.

A significant formation of amyloid β plaques and neurofibrillary tangles in the hippocampus was found in aged female 3xTg-AD mice (Carroll et al., 2007). OVX-induced E2 depletion in female 3xTg-AD mice further increased amyloid β plaques, but E2 supplementation reduced these amyloid β plaques. Interestingly, female 3xTg-AD mice showed greater Aβ plaques and larger behavioral deficits than male 3xTg-AD mice (Carroll et al., 2010).

Impairment of LTP in AD model animals was investigated, using OVX female rats treated with intraperitoneal injection of amyloid β (Shang et al., 2010). LTP measurements in CA1 were performed in acute hippocampal slices prepared from rats treated with amyloid β injection. CA1-LTP showed impairment by amyloid β injection. Furthermore, no impairment on CA1-LTP was observed in hippocampal slices prepared from E2-supplemented (with E2-pellet) OVX rats treated with amyloid β injection (Shang et al., 2010).

In summary, nE2 plays an important role in protection against cognitive decline in not only normal aging but also AD-related diseases.

## Glossary

AD: Alzheimer’s disease
AMS: Aging males’ symptoms scale
AR: Androgen receptor
BDNF: Brain-derived neurotrophic factor
cir-E2: Circulating estradiol
cir-PROG: Circulating progesterone
cir-T: Circulating testosterone
ER: Estrogen receptor
EPSP: Excitatory postsynaptic potential
EPSC: Excitatory postsynaptic current
FBN-ARO-KO: Forebrain-neuron-specific aromatase knock-out
IIEF-5: International index of erectile function score
LOH: Late-onset hypogonadism
LTP: Long-term potentiation
LTD: Long-term depression
mAR: Membrane AR
mERα: Membrane ERα
mERβ: Membrane ERβ
NGF: Nerve growth factor
NMDA: N-methyl-D-aspartic acid
nAllo: Neuro-allopregnanolone
nDHT: Neuro-dihydrotestosterone
nE2: Neuro-estradiol
nPROG: Neuro-progesterone
nT: Neuro-testosterone
nERα: Nuclear ERα
nERβ: Nuclear ERβ
OVX: Ovariectomized
PFC: Prefrontal cortex
P450arom: Cytochrome P450 aromatase
P450(17α): P450 17α hydroxylase
SAMP8: Senescence accelerated mouse-prone 8
SDS: Self-rating depression scale
TP: Testosterone propionate
TRT: Testosterone replacement therapy
17β-HSD: 17β-hydroxysteroid dehydrogenase
3m: 3-month-old
12m: 12-month-old
24m: 24-month-old
