# Supplementary material for: Local synthesis and function of neuro-estrogen and neuro-androgen: impact on the hippocampus-related cognition and neuronal plasticity
Source: Front Cell Neurosci. 2026 Jan 28;19:1695565. doi: 10.3389/fncel.2025.1695565 (PMC12892977; doi:10.3389/fncel.2025.1695565)
Supplement: Supplementary file 1 [file Supplementary_file_1.pdf]

Supplementary Figures

Supplementary Figure S1

Neuro-androgen (nT and nDHT) synthesis in adult female and male rat hippocampus. (A) Comparison of hippocampal level of androgens (nT and nDHT) between adult female and male rats, measured with LC-MS/MS. Hippocampal androgen level was significantly different between female and male. The levels of nT and nDHT are expressed in nM (left axis) and ng/g wet weight of hippocampal tissue (right axis). (B) Good correlation is shown between hippocampal T and DHT for individual rats. Data for male were taken from (Hojo et al 2009) and data for female from (Hojo et al 2014). (C) Typical HPLC profiles of ^{3}H-T metabolites in female hippocampus. nDHT, nE2 and androstanediol (DHT metabolite) are produced. (D) Comparison of hippocampal allopregnanolone (Allo) level between male and female. Hippocampal level of Allo is higher in female than male.

Figures are modified from (Hojo et al 2014) (Hojo & Kawato 2018))

Supplementary Figure S1

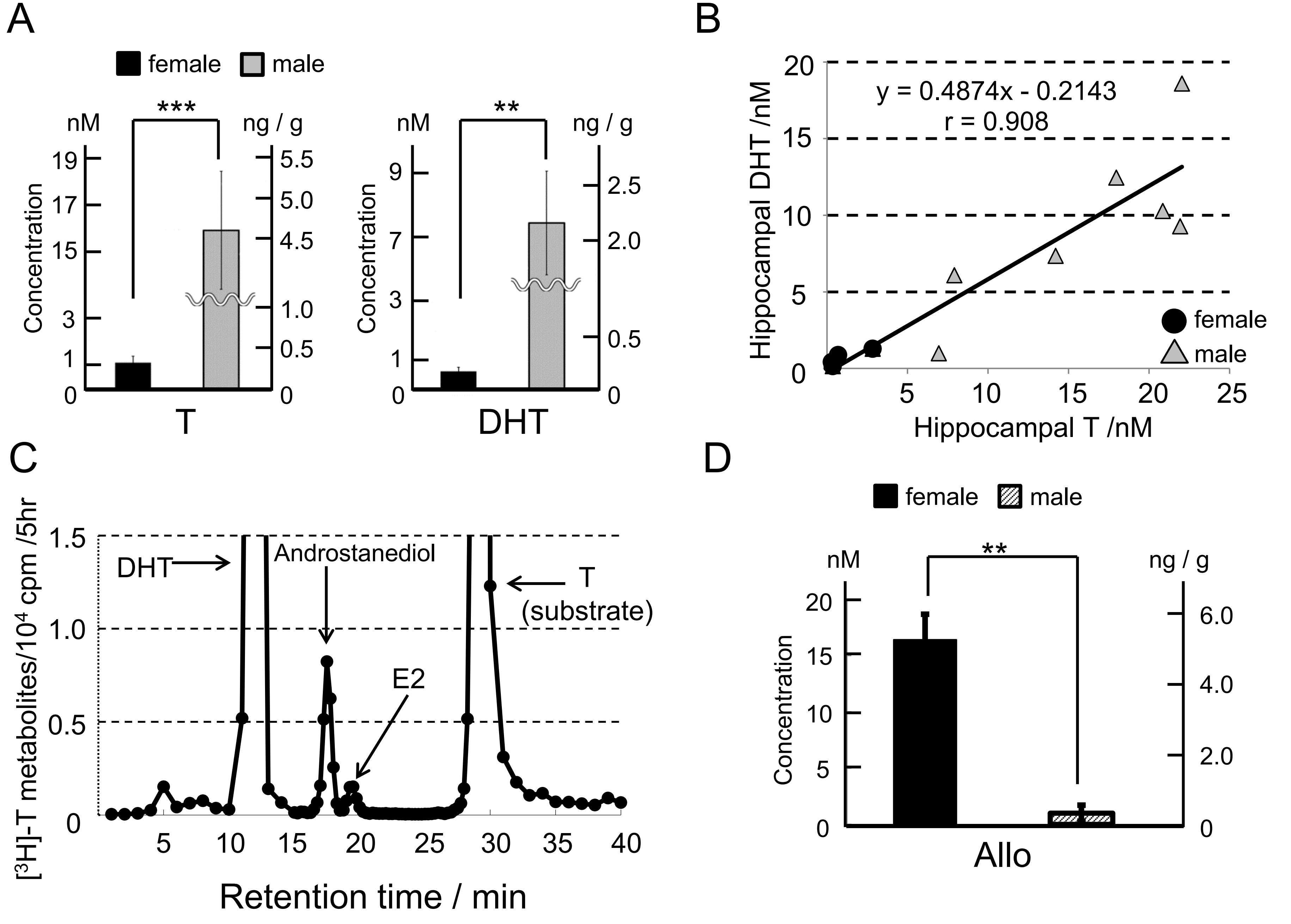

Supplementary Figure S2

Comparison between male and female about mRNA expression levels of steroidogenic enzymes and receptors in adult rat hippocampus. Steroidogenic enzymes are P450(17α), P450arom, 17β-HSD1, 17β-HSD3, 5α-reductase 1, 5α-reductase 2 and Star. These enzymes contribute to synthesis and action of nE2, nT and nDHT. Steroid receptors are ERα, ERβ and AR.

In female, no estrus cycle dependent changes are observed for these enzymes and receptors.

Representative gel images for PCR products (Upper), and relative expression levels of each mRNA (Lower) are shown. In each image, from left to right, size marker (100 bp ladder) (M), male hippocampus (male), female hippocampus at proestrus (P), estrus (E), diestrus 1 (D1), diestrus 2 (D2), and OVX female rats (OVX). Negative control (Nc) without template DNA and Positive control (Liver (Liv), prostate (Pro) and adrenal gland (Adr)). *Gapdh* (GAPDH) is an internal control.

Figures are modified from (Kato et al 2013) (Hojo et al 2014).

Supplementary Figure S2

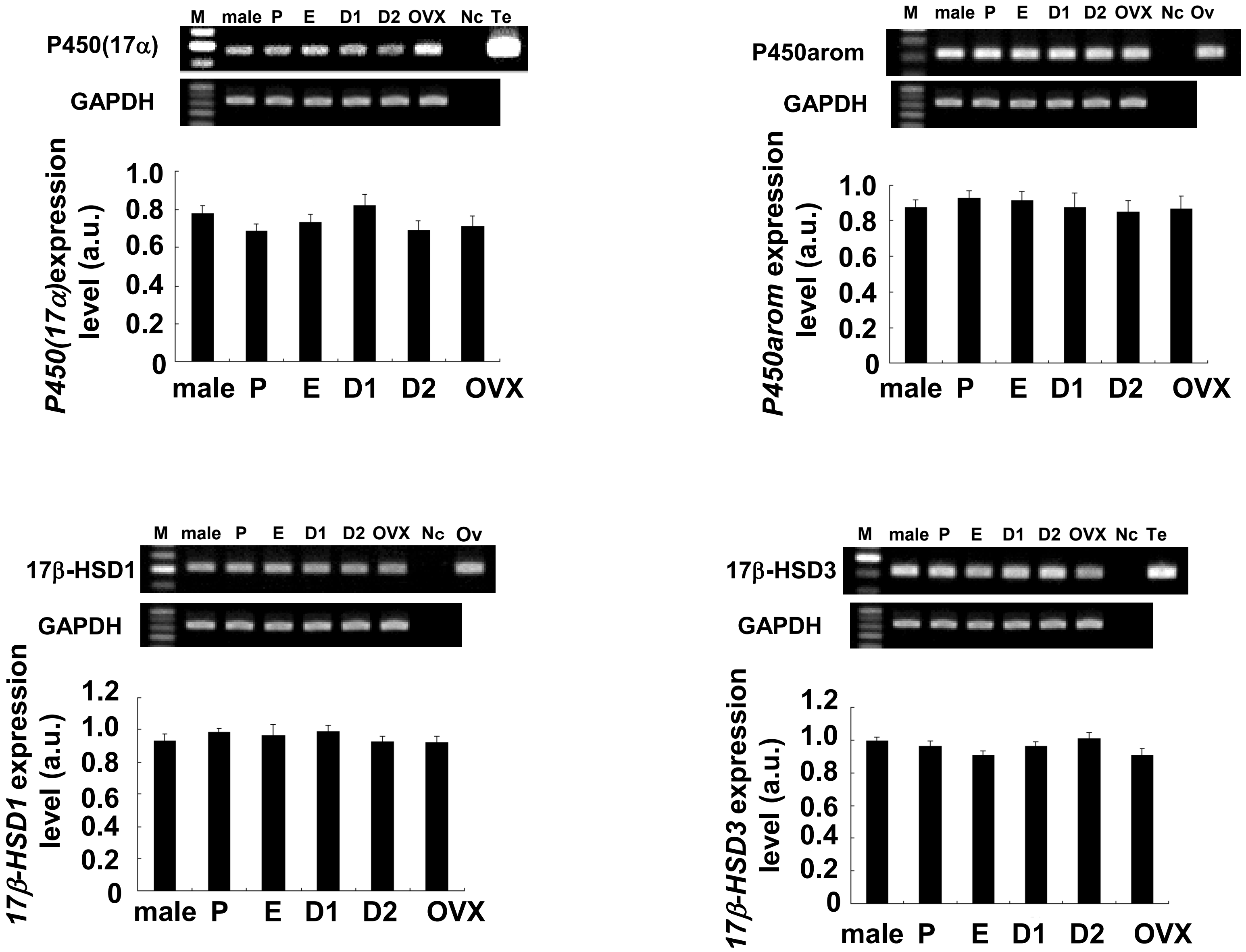

Supplementary Figure S2

Srd5a1
(5α-reductase1)

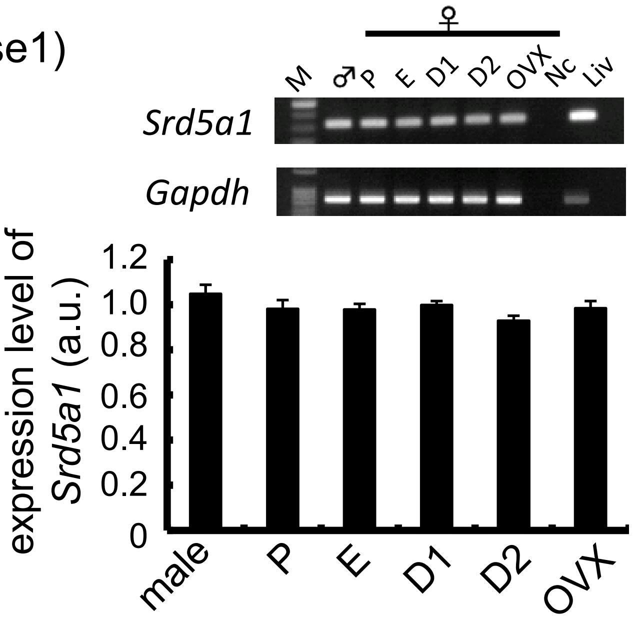

Srd5a2
(5α-reductase2)

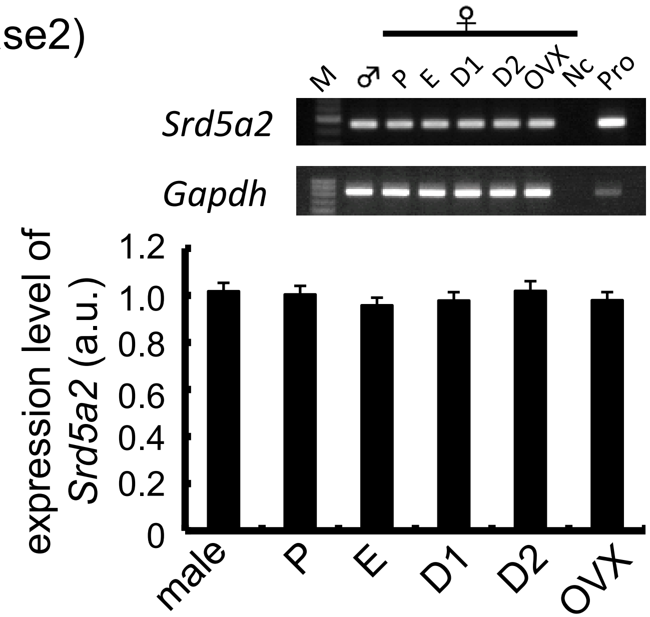

Star

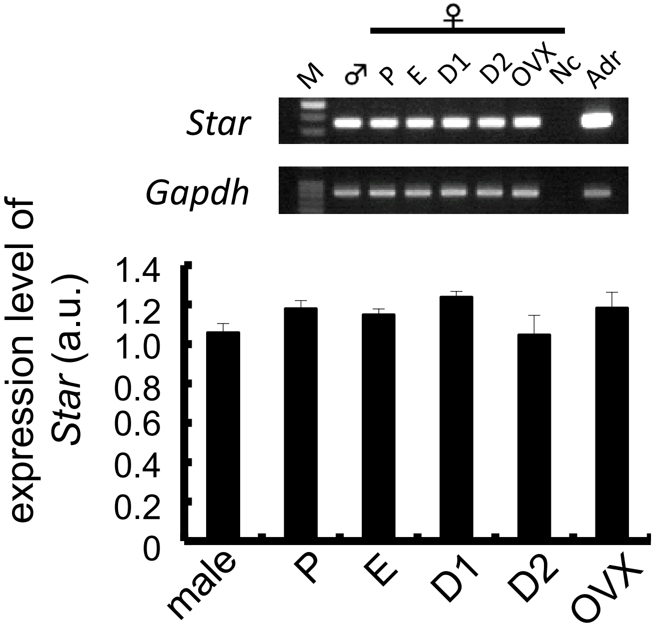

Supplementary Figure S2

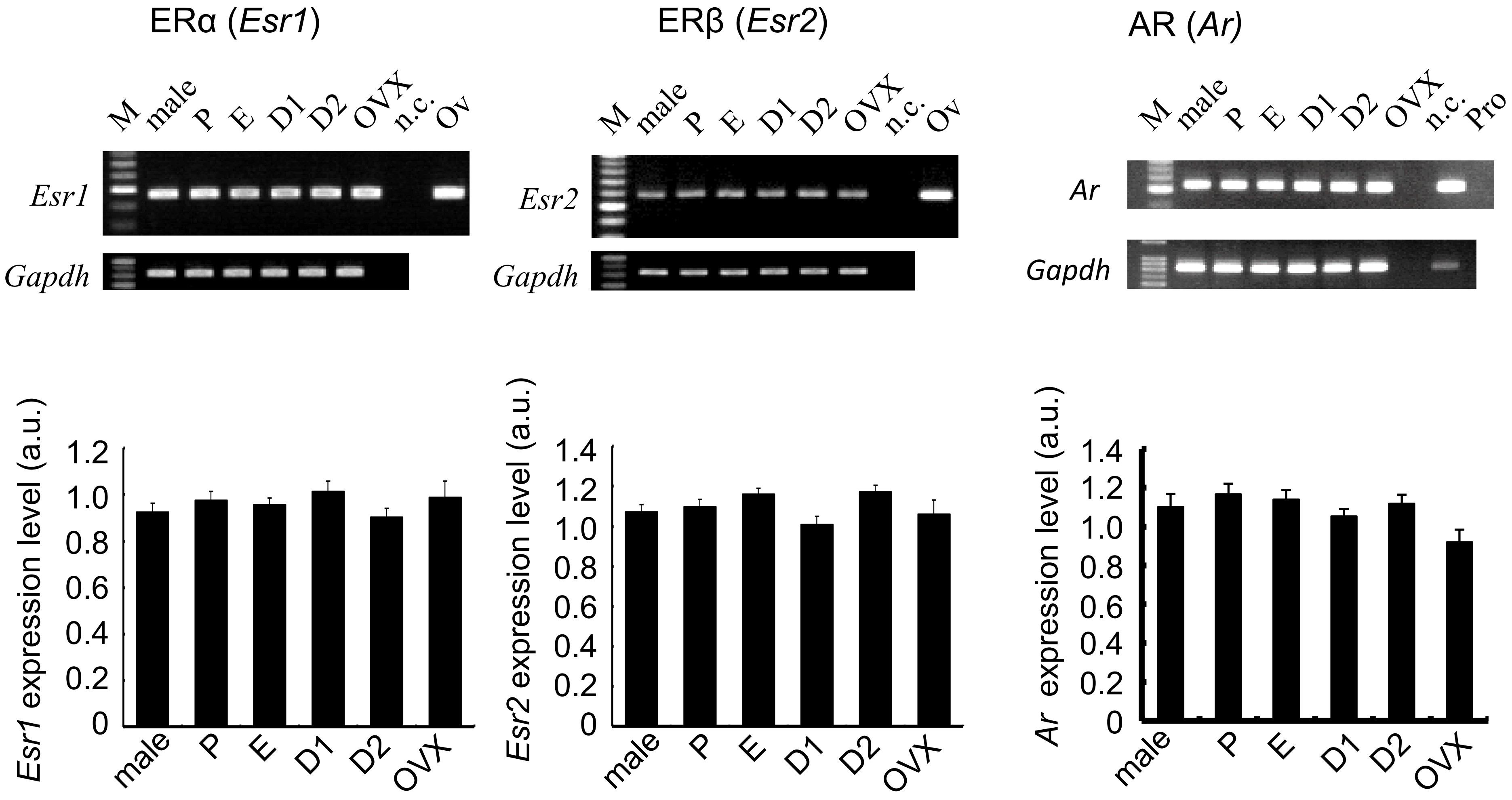

Supplementary Figure S3

Comparison between female and male about immunostaining of steroidogenic enzymes (A) and steroid receptors (B) in adult rat hippocampus. Adult female rat hippocampus at Proestrus stage is shown. Scale bar, 800 μm. (A) Steroidogenic enzymes, including , StAR, P450scc, P450(17α), P450arom and 3β-HSD, localized in the glutamatergic neurons (in CA1, CA3 and DG). (B) Receptors for sex steroids including AR and ERα, localized in the glutamatergic neurons in CA1, CA3 and DG.

Figures are modified from (Kimoto et al 2001) (Shibuya et al 2003) (Hojo et al 2004) (Hojo et al 2008) (Mukai et al 2010) (Hojo et al 2014).

(A) Steroidogenic Enzymes

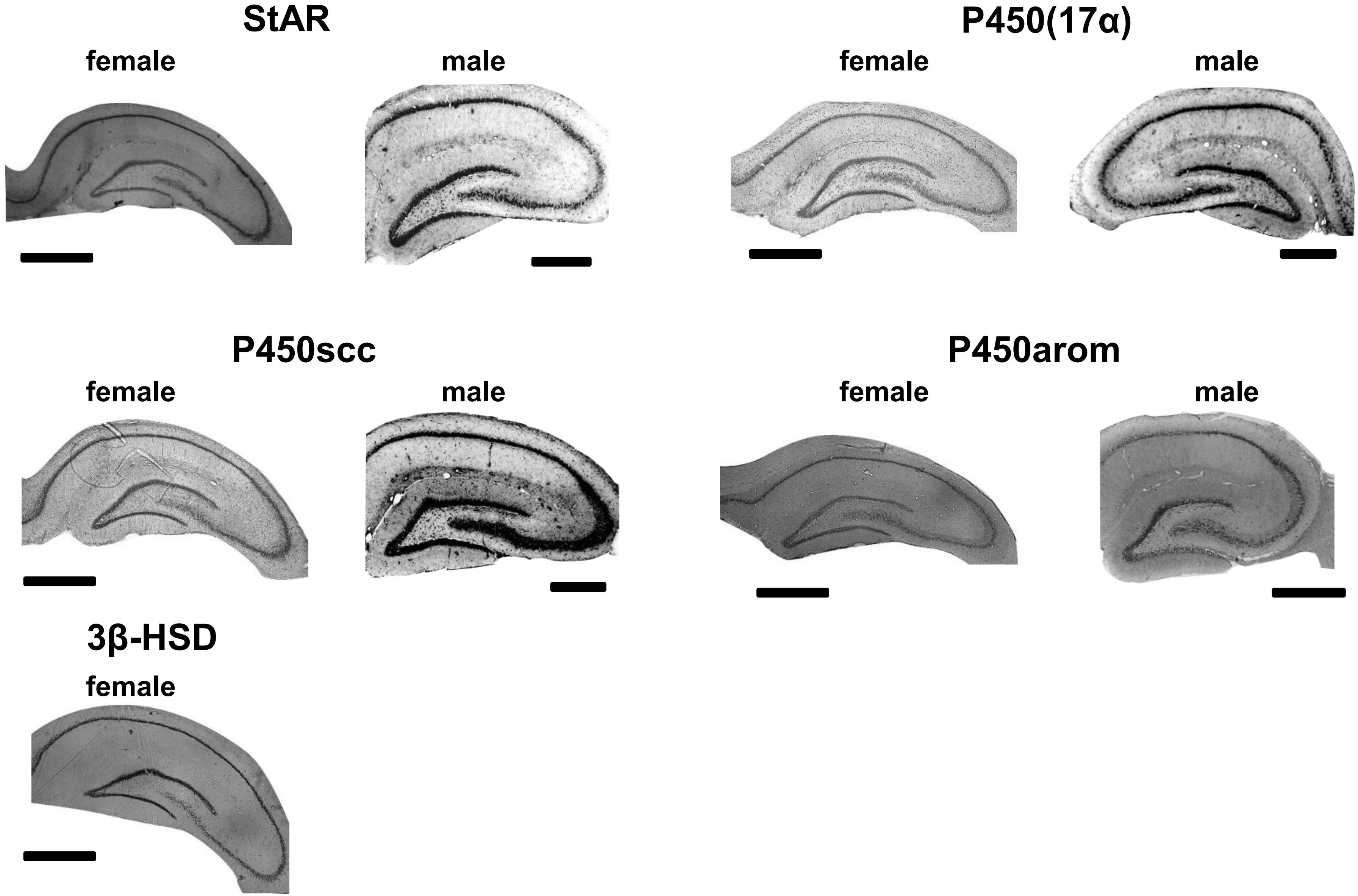

(B) Steroid Receptors

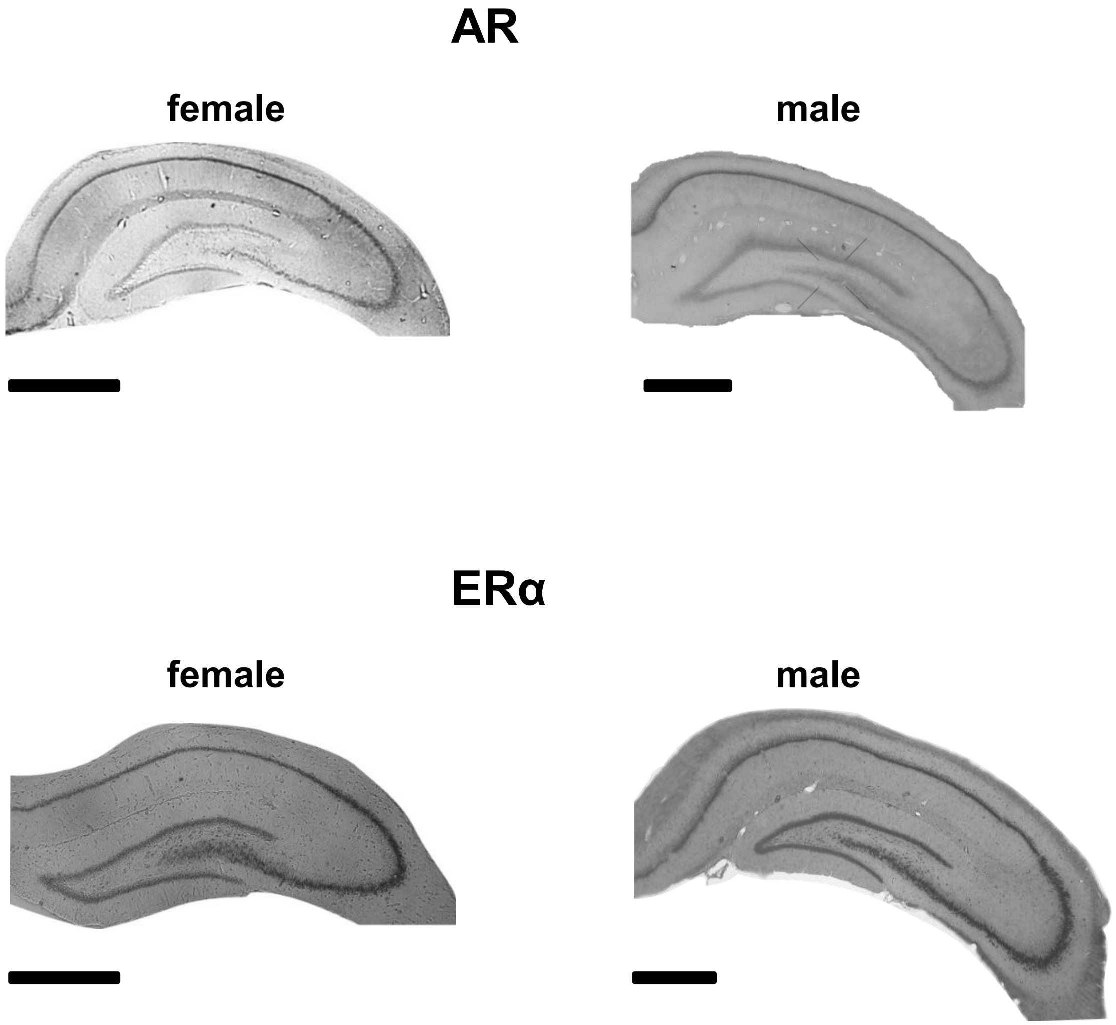

References for Supplementary Figures

Hojo Y, Hattori TA, Enami T, Furukawa A, Suzuki K, et al. 2004. Adult male rat hippocampus synthesizes estradiol from pregnenolone by cytochromes P45017alpha and P450 aromatase localized in neurons. *Proc Natl Acad Sci U S A* 101: 865-70

Hojo Y, Kawato S. 2018. Neurosteroids in Adult Hippocampus of Male and Female Rodents: Biosynthesis and Actions of Sex Steroids. *Front Endocrinol (Lausanne)* 9: 183

Hojo Y, Murakami G, Mukai H, Higo S, Hatanaka Y, et al. 2008. Estrogen synthesis in the brain--role in synaptic plasticity and memory. *Mol Cell Endocrinol* 290: 31-43

Hojo Y, Okamoto M, Kato A, Higo S, Sakai F, et al. 2014. Neurosteroid synthesis in adult female rat hippocampus, including androgens and allopregnanolone. *J Steroids and Hormonal Sci*: Article S4

Kato A, Hojo Y, Higo S, Komatsuzaki Y, Murakami G, et al. 2013. Female hippocampal estrogens have a significant correlation with cyclic fluctuation of hippocampal spines. *Front Neural Circuits* 7: 149

Kimoto T, Tsurugizawa T, Ohta Y, Makino J, Tamura H, et al. 2001. Neurosteroid synthesis by cytochrome p450-containing systems localized in the rat brain hippocampal neurons: N-methyl-D-aspartate and calcium-dependent synthesis. *Endocrinology* 142: 3578-89

Mukai H, Kimoto T, Hojo Y, Kawato S, Murakami G, et al. 2010. Modulation of synaptic plasticity by brain estrogen in the hippocampus. *Biochim Biophys Acta* 1800: 1030-44

Shibuya K, Takata N, Hojo Y, Furukawa A, Yasumatsu N, et al. 2003. Hippocampal cytochrome P450s synthesize brain neurosteroids which are paracrine neuromodulators of synaptic signal transduction. *Biochim Biophys Acta* 1619: 301-16
